# Naturalizing Darwall’s Second Person Standpoint

**DOI:** 10.1007/s12124-020-09547-y

**Published:** 2020-05-27

**Authors:** Carme Isern-Mas, Antoni Gomila

**Affiliations:** Human Evolution and Cognition Group (EvoCog), Associated Unit to CSIC, Laboratori de Sistemàtica Humana, UIB, IFISC, University of the Balearic Islands, Campus Carretera Valldemossa, 07122 Palma de Mallorca, Spain

**Keywords:** Second-person, Moral motivation, Special obligations, Moral emotions, Moral community, Group norms

## Abstract

In this paper, we take Darwall’s analytical project of the second-person standpoint as the starting point for a naturalistic project about our moral psychology. In his project, Darwall contends that our moral notions constitutively imply the perspective of second-personal interaction, i.e. the interaction of two mutually recognized agents who make and acknowledge claims on one another. This allows him to explain the distinctive purported authority of morality. Yet a naturalized interpretation of it has potential as an account of our moral psychology. We propose a naturalistic interpretation of Darwall’s work to address some of the main issues about our moral psychology. First, we explain why moral norms motivate us; namely, because of these second-personal relations. We provide a naturalized version of this solution. Second, we articulate how intersubjective interactions take place effectively; grounding duties to particular other subjects, and being related to distinctive moral emotions. Third, we address the question of the limits of the moral community, proposing that it comprises all agents capable of second-personal interactions. Finally, we explain the emergence of community norms through intersubjective interaction. Since all group members can adopt alternatively the second-personal stance to each other, demands are sanctioned and recognized in a triangulation process which explains the emergence of group norms.

## Introduction

There have been several attempts to ground morality in the way we relate to others. First attempts in this line can be found in the Idealist school. In reaction to Kant’s abstract deontology, authors such as Fichte (1797/2000), or Hegel (1807/1979), formulated the idea that mutual recognition sets up the subject as a subject first, and as a subject of rights, next. To become a self, one needs to confront other selves. Thus, their focus was on the process of constitution of a subject, trying to spot the relevant interactions to this extent. In the 20th century, in contrast, the most salient attempts at developing this intersubjective approach, such as Ricoeur’s (1954) or Lévinas’ (1969), were of phenomenological character. They contended that some basic human experiences, such as sympathy or compassion, or even eye contact, already involve a normative dimension. In this phenomenological tradition the subject is prior to the interaction with an ‘other’, and the project is to describe what appears in the conscious experience, even if it does not exhaust the alterity of the other. Hence, reciprocity and interaction are considered as they are experienced (Gomila 2001a). Besides, and more importantly, these authors try to derive universal duties out of these experiences and ground them in privileged forms of intersubjectivity. However, it is not clear that a particular kind of intersubjective relationship grants the recognition of these duties (Gomila 2008).

Stephen Darwall’s project of a ‘second-personal morality’ (Darwall 2006, 2013a, b) is something different, even if it also takes intersubjective interaction as a touchstone. To begin with, it is analytical and normative, rather than descriptive or explanatory. It aims to show that moral notions, such as duty and obligation, constitutively imply our second-personal interaction with others. In a second-personal interaction a subject addresses a claim or demand to another, who can recognize the claim or demand as valid or not. And through these interactive dynamics of claims, recognitions, mutual demands and reasons, both subjects hold each other accountable. According to Darwall, it is this holding each other accountable that is implicit in the moral notions, such as respect or dignity. Similarly, this second-personal network of accountability and recognition justifies the Kantian formal principle of normative universalization.

Darwall’s proposal does not delve into the nature of our actual interpersonal relationships. As we have said, his proposal is normative at heart. He draws from rational and free agents in a kind of interaction, second-personal interaction, which does not actually need to take place. However, if we draw from flesh and blood subjects in their particular interpersonal relationships, that is, subjects who bond with each other, we can give a more accurate account of our moral psychology. In this paper, we make explicit the naturalistic dimension presupposed by Darwall’s theory, and use it to provide an account of our moral psychology.

In "The Second-person Standpoint as a Conceptual Analysis" section, we describe Darwall’s analytical project of connecting intersubjectivity and morality. In "The Motivational Power of Moral Judgments" section, we contend that this connection presupposes a naturalistic articulation. This naturalistic approach allows us to address some of the main questions in moral psychology, and meta-ethics. We provide a naturalistic account of the motivational power of moral judgments, in "Special Obligations Towards Particular Subjects" section, and of our special obligations to particular subjects, in "Moral Emotions" section; both based on intersubjective interactions. In "The Limits of the Moral Community" section, we show how intersubjective interactions are already to be found in moral emotions. In "The Emergence of Group Norms" section, we tackle the question of the limits of the moral community, proposing that it comprises all agents capable of second-personal interactions. And, finally, in "Conclusion" section, we explain the emergence of community norms through intersubjective interaction. Needless to say, our project is not to propose a re-interpretation of Darwall’s work, which is neither descriptive nor naturalistic, but rather an original account of our moral psychology inspired in his work.

## The Second-person Standpoint as a Conceptual Analysis

Darwall defines the second-person standpoint as “the perspective you and I take up when we make and acknowledge claims on one another’s conduct and will” (Darwall 2006, p. 3); the perspective we take in the practices of holding each other accountable and responding to those claims. According to Darwall, these second-personal practices are relevant for morality because moral notions involve second-personal notions, and because the grounds of moral motivation lie in the second-personal relationship. Moral notions do not stand in a rational heaven, but presuppose those second-personal practices among moral subjects.

To account for the second-person standpoint, Darwall proposes the following situation as the paradigmatic instance of a second-personal interaction. The interaction starts when one person steps on another’s foot. Hereafter, the person who steps on the other’s foot will be the “transgressor”, and the person whose foot is stepped onto will be the “victim”. Being persons, and hence having equal dignity, they both have the authority to demand a certain treatment of each other. Furthermore, as their relation is governed by what Darwall calls “reciprocal recognition” (Darwall 2006, p. 48), they both recognize each other and can address demands to each other. Accordingly, the victim demands the transgressor to move his foot; and the transgressor knows and feels that they ought to accept and respect the victim’s claim. This feeling comes from the second-personal nature of the relationship at issue: it is a relationship of reciprocal accountability through which they address demands to each other, and hold one another responsible for compliance. At the same time, the victim reacts to the transgressor’s reaction, accepts their stepping behind, and the relationship is reestablished. In this way, the problem of moral motivation finds another, more promising answer: the purported authority of morality, i.e. its motivational power, derives from this recognition of others as sources of obligation.

Notice that Darwall’s account is analytical in the first place. It does not describe what is going on in cases of harming one another, but claims to unpack the content of the notions that characterize morality. In other words, morality constitutively requires that these patterns of mutual recognition, of addressing claims and honoring them, take place. In this way, the account also becomes normative in that at the same time it sets the standards for real world human interactions to count as properly moral. Mutual respect becomes mandatory for moral agents as long as it is implicit in the very notion of morality.

This analytical and normative stance entails that Darwall does not want to accept the consequence that moral obligation derives from mutual demands. According to him, stepping on the victim’s foot is wrong even if the victim does not protest. In fact, the transgressor’s feeling that they ought to respect their victim’s claim and move their foot comes from the transgressor’s knowing that they could justifiably be held accountable for incompliance, even by themselves in their own conscience, as if they adopted a second-person standpoint towards themselves. If the transgressor did not move their foot, they would be accountable to their victim’s claim for respect of their dignity as a person, even if the victim did not make it. Consequently, the transgressor accepts the victim’s right to claim, and reacts to it by moving their foot.

In this pattern of interaction, several assumptions and concepts are in play, according to Darwall. To make them clear, we will start with the assumptions; and move next to the concepts, which constitute an “interdefinable circle” (Darwall 2006, p. 12) where each one implies all the rest. Hence the analytical nature of the approach. Let us disentangle each assumption and concept one after the other, and see how they relate.

In the practice of holding accountable, i.e. of giving and asking for reasons, agents involved in an interaction assume that they both have: (1) a right to make claims, to demand respect for those claims, and to resist the demands of the other; (2) a second-personal authority to make demands or claims, and to hold the other accountable for non-compliance without excuse; and, (3) a dignity which must be respected and which cannot be violated by any claim. As this characterization makes clear, Darwall’s second-personal standpoint is normative, rather than descriptive. It specifies a desideratum, rather than describes how things always happen. The circle of concepts specifies the way in which particular interactions should take place to qualify as properly moral.

One of those concepts is second-personal authority. Second-personal authority is the authority that a moral subject has to address claims, and demands to other subjects. For the addressee, the claim creates a distinctive reason for compliance. In Darwall’s paradigmatic example, both the transgressor and the victim have authority to address demands to each other. Furthermore, the victim’s demand that the transgressor move their foot makes the transgressor responsible for complying, since the transgressor recognizes the practical authority of their victim. This practical authority is presupposed when an addresser claims or demands something of an addressee, and this addressee recognizes the addresser’s right to so claim. Therefore, the concept of authority is necessarily tied to other second-personal concepts. First, second-personal authority is second-personal because it assumes that it is addressed to particular subjects in interaction. Second, it entails second-personal competence, which means that “whenever second-personal address asserts or presupposes differential authority, it must assume also that this authority is acceptable to its addressee simply as a free and rational agent” (Darwall 2006, p. 22). Besides, the addresser must also assume the addressee’s capacity of free self-determination to accept internally the authoritative demand, and decide whether or not to respond to it. Finally, the notion of second-personal authority involves necessarily the notion of responsibility or accountability. The authority to demand implies not just a reason for the addressee to comply, but also their being responsible for doing so and their accepting the possibility of being held accountable for non-compliance without excuse.

As for second-personal responsibility, it “concerns how, in light of what someone has done, she is to be related to, that is, regarded and addressed (including herself) within the second-personal relationship we stand in as members of the moral community” (Darwall 2006, p. 69). We see this notion graphically illustrated in daily situations such as those in which a caretaker scolds a child for something they has just done. In most of these cases, the caretaker points at a drawing in the wall, or a messy table, and yells at the child “look what you have done”. Somehow, this caretaker is trying to make explicit the relation of the child to what they has done, and to hold them accountable for it. Indeed, Darwall understands responsibility as accountability; in other words, we are responsible for what a member of the moral community can hold us accountable for doing (again, even if nobody never does).

What invests agents with authority is the fact that they can address claims to each other. Such claims provide reasons both explicitly through speech acts, such as reproaches, excuses, or requests; or implicitly through reactive attitudes such as resentment, indignation or anger. For instance, the person whose foot is stepped onto can either verbally ask the transgressor to move their foot, and hence address explicitly a reason through a speech act; or make a gesture of protest showing their disapproval, expressing resentment at the transgressor’s action, and hence addressing the reason implicitly through a reactive attitude. These reactive attitudes are forms of address that directly appeal to the addressee’s goodwill and that hold him accountable for compliance.

In these dynamics of second-personal interaction, as spelled out by Darwall, what is given through a demand is a second-personal reason. The victim’s resentment at the transgressor’s stepping onto their foot counts as a reason for the transgressor to move their foot. This reason is second-personal because it has the following features. First, it is an agent-relative practical reason; that is, it is a reason for acting “whose validity depends on presupposed authority and accountability relations between persons and, therefore, on the possibility of the reason’s being addressed person-to-person” (Darwall 2006, p. 8). Second, it aims at motivating the other’s will through the agent’s own self-determining choice. Accordingly, it is not a kind of coercion, but an internal acceptance of an authoritative demand. In Darwall’s example, the victim seeks compliance after recognition, instead of mere obedience from the transgressor. Third, and as a result of being part of the dynamics of the second-person standpoint, second-personal reasons presuppose that both agents have equally second-personal authority, competence, and responsibility as free and rational agents, and that they can exchange their positions as addresser and addressee.

In summary, according to Darwall the validity of second-personal reasons depends on the authority and accountability relations between addressees and addressers, who can exchange their roles in their interaction; and on the ability of the participants to self-determine themselves freely by acknowledging the authority of the other agents they interact with. However, Darwall avoids the conclusion that claims are justified if they are addressed, or accepted. According to Darwall, the justification of those claims is established independently, in so far as they are universalizable, à la Kant. That is why he insists that the demands can be internally recognized, as if the second-person standpoint was internalized. On the contrary, a naturalistic approach assumes that the sort of impersonal point of view required to establish the universalizability of claims the analytical approach requires does not exist. As a consequence, the legitimacy of moral norms and practices of mutual recognition has to be viewed as grounded in the dynamics of intersubjective demands.

## A Naturalistic Approach to the Second-person

So far we have introduced the basics of Darwall’s analytical project. In what follows, we try to develop it into a naturalistic framework, as we think it provides useful elements for an account of our moral psychology. As we have already remarked, Darwall’s approach is presented as analytical. Yet, as a matter of fact, it turns out to be normative, as it assumes rational and free agents, and takes the normative dimension as universal and independent of the particular claims agents address to each other. Darwall’s model is inspired by contractualism at heart: rational subjects recognize each other as such, giving rise to mutual respect, reciprocal claiming, and deals. All rational subjects are interchangeable, and they have internalized this second-personal standpoint: their moral conscience results from this self-assessment according to already legitimate demands. However, the sort of second-personal interaction that Darwall describes also invites another way to develop it: as a naturalistic account of how such dynamics of claims and respect for them takes place, and how the remarkable kind of moral agents can emerge in the first place; how such subjects are constituted by the sort of intersubjective interactions described; and to what extent we humans can approach such normative ideal of rationality.

For instance, Darwall’s free and rational agents need to be endowed with a full set of psychological capacities. They have to be linguistic beings to verbally address and receive claims. They also have to be emotional beings, to address claims implicitly through their reactive attitudes. They also require some degree of self-control and self-regulation, to behave in a self-initiated way. And they need to understand others, through some form of psychological attribution, to recognize others’ intentions, plans, and emotions, and respond to them. Maybe also some kind of empathy, compassion, or sympathy needs to be presupposed.

Thus, although Darwall’s project is formulated as analytical, it also calls for a naturalistic project, which accounts for flesh and blood subjects who are somehow sensitive to others’ demands, and who need affectively bonding with others. Hence, in our view Darwall’s proposal requires a naturalistic counterpart, and offers the seeds for it, even if Darwall is not interested in such a project. Similarly, an appropriate naturalistic project can benefit from his characterization of morality as intrinsically second-personal, to articulate the way in which our moral psychology is shaped by second-person interaction.

In this way, Darwall’s second person standpoint presupposes a more basic notion of second personal interaction, as the way we come to interact with particular others, before and beyond accountability –an approach we have tried to independently develop (Gomila 2002, 2008, 2015). Instead of characterizing the second-person perspective as intrinsically moral, the naturalistic approach focuses on how the intersubjective structure of recognition emerges in interaction. In this view, the second-person perspective is the way in which we attribute mutually, and implicitly, expressive mental states, such as intentions and emotions, to those with whom we interact face to face (Gomila 2001a, 2002, 2015). It is our spontaneous way to make sense and adjust to others’ behavior in face to face interactions. From this point of view, the second-person perspective characterizes the psychological competence which ensures mutual understanding in intersubjective interaction (Gomila 2008).

In this naturalistic project, morality is still grounded in the second-person perspective because morality requires our ability to interact with others in an intersubjective way (Gomila 2008). We need to attribute intentions, keep track of epistemic states and recognize emotional expressions, if we are to make sense of the claims and demands that others may address to us, and respond properly. Think, for instance, of Strawson’s reactive attitudes, which Darwall takes as a case of implicit second-personal claim addressed to another. To react with resentment to another’s deeds, we need to see their behavior as intentional in the first place, and with a particular intention, given the context. Thus, Darwall’s normative project presupposes our naturalistic project of the second-person as the perspective of intentional interaction in real time. Furthermore, in Darwall’s project agents need not simply to attribute or recognize an emotion in their interactive party in a distanced, off-line, third personal way; but to react emotionally and intentionally to them in an engaged, online, reciprocally contingent, second-personal way (Gomila 2002). Darwall does not consider in detail the complexities of psychological attributions, and just assumes the view of simulation theory (Goldman 1992; Gordon 1992).

This naturalistic understanding of the second-person standpoint can account for our moral psychology. In what follows, we use our naturalization of Darwall’s project to put forward five essential issues in moral psychology: the motivational power of moral judgments; special obligations we recognize towards particular subjects; the intersubjectivity involved in moral emotions; the limits of the moral community; and the emergence of moral group norms.

## The Motivational Power of Moral Judgments

One of the topics of moral psychology which can be addressed from this naturalized view of the second-person standpoint of morality is the phenomenon of moral motivation. Moral motivation is the phenomenon by which we feel somehow motivated to act in accordance with our moral judgments (Rosati 2016). For instance, it is what makes us feel obliged to help friends in need. It is not just that we judge that it is morally correct to help them by applying some general norm to the situation in question. For if moral reasoning were like that, our moral judgment would be similar to our judgment to drive on the left side when we are in Great Britain: this is the correct thing to do in the situation, but relative to a context. It would be a sort of prudential judgment, based on the willingness to comply with the established norms and on the fear of the negative consequences of not doing so. But moral judgments are different. Moral judgments motivate in a distinctive way: we feel obliged to comply with them. This sense of obligation can involve several aspects: a bodily sense of urgency, an anticipation of shame at the thought of failing to comply, remorse and lower self-esteem if failure did happen. Morality contributes to our identities (Riis et al. 2008; Strohminger and Nichols 2014, 2015; Tobia 2015).

For instance, in Victor Hugo’s *Les Misérables*, when Marius recognizes that he ought to stay at the barricade and fight with his colleagues instead of running after his beloved Cosette, he does not just recognize his duty, he also feels that he cannot but comply with it. He feels motivated to do what he thinks his duty is. Not only does he judge that fighting is his duty, but he also feels unease at the idea of running after Cosette. He anticipates a sense of cowardice and treachery if he were to escape from the fight to go after his beloved. His decision to stay at the barricade is not just a deliberate decision out of a prudential calculation of pros and cons, of gains and losses. It is not the choice of the most optimal benefits. It is neither the result of the application of a general principle to a particular case. It is the consequence of his feeling the urge to stay, to comply with his duty because failing to do so would amount to reveal an evil moral identity.

Moral judgments, then, have this dual character. On the one hand, they are not just a matter of taste, or of personal inclination; they are truth-apt, as moral cognitivists would emphasize. For instance, fighting at the barricades is felt by Marius as the right action for anyone in a similar position. It seems to be something externally imposed, a truth to be recognized, maybe learned. But at the same time, moral judgments are experienced psychologically in a way that descriptive statements do not: they involve emotions about oneself and one’s sense of self. They are strongly related to motivation, as moral non-cognitivists notice. They are experienced as a subjective commitment. Thus, before the meta-ethical debate about their ontology, what we can claim from our naturalistic view is that moral judgments are psychologically experienced both as objective and subjective (Isern-Mas and Gomila 2018).

From the second-person standpoint, the motivational power of moral judgments can be explained by their second-personal character. As we have mentioned, according to Darwall, we feel obliged to follow our moral judgments because they imply the recognition of the legitimacy of the claim of another agent. Remember the case of the person whose feet the transgressor stepped onto. The victim has a second-personal authority to hold the transgressor accountable if they rejected to move their foot. By respecting the addresser’s claim right and second-personal authority, the addressee, i.e. the transgressor, is responsible for compliance and must be prepared to be held accountable if they does not comply. And it is this knowing that they could justifiably be held accountable which motivates the transgressor to comply with the demands that other members can legitimately address to them, i.e., their moral obligations, according to Darwall.

Therefore, moral judgment has motivational power on us because it is essentially interpersonal, intersubjective or, in Darwall’s terms, second-personal. For instance, I feel, and know, that I ought to help my friends in need, because this is what them, or any other members of the moral community including myself, could demand me to do; or could hold me accountable for not doing. Likewise, I ought not to mistreat my partner, because if I did it, he would have the right to hold me accountable for doing it, and I should blame myself too. Accordingly, the motivational power of moral judgment does not come from our being aware of the moral law through moral deliberation, *à la* Kant, or from a calculation of consequences, as utilitarianism prescribes. It comes from the motivational nature of second-personal claims and reasons; which motivate us because they come from a recognized authority, so that they become internalized. In fact, moral obligations are defined as “what those to whom we are morally responsible have the authority to demand that we do” (Darwall 2006, p. 14), or “what the moral community can demand (and what no one has the right not to do)” (Darwall 2006, p. 20). Therefore, it is because we assume as our own (at least some of) others’ moral demands: those that we honor and find justified. Therefore, the motivational power of morality derives from our receptiveness to the demands of those with whom we interact (Isern-Mas and Gomila 2018).

All this structure need not to be explicit, as Darwall himself recognizes. The Strawsonian reactive attitudes (Strawson 1974) implicitly involve this web of reciprocal expectations. For instance, my knowing that my friends could hold me accountable for not helping them is manifested in my feelings of guilt, which is just “to feel as it one has the requisite capacity and standing to be addressed as responsible” (Darwall 2006, p. 71). This emotion-laden interaction is what makes us feel bound by others’ demands on us, and eventually by the moral norms which will emerge from those interactions. Indeed, according to Carla Bagnoli, the “apparent inescapability of moral norms and the specific kind of authority that they have in our minds” is due to moral emotions (2006, p. 8). Therefore, it is through emotions that we feel motivated to act according to our moral judgments.

If we had no moral sensibility we would have only extrinsic motives to enforce moral norms, such as sanctions and incentives, fear of punishment and expectation of reward. We are able to undertake morality as a subjective motive because we are capable of moral sensibility. (Bagnoli 2006, p. 13)

The naturalistic twist can offer an account of why we are so susceptible to each other’s demands. The answer has to do with the fact that we are a social or, more precisely, an “ultra-social” species (Tomasello 1999, p. 59). Our evolutionary origins make us feel motivated to bond with others and to take their interests and needs into account (Cheney and Seyfarth 2008; Seyfarth and Cheney 2012; Tomasello 2016). Within this evolutionary path of interpersonal dependencies, morality seems to have emerged as a game of reciprocity requests that are recognized and self-imposed, instead of imposed through coercion, or fear of punishment. Evolution made us pro-social in the first place; and morally motivated afterwards.

## Special Obligations Towards Particular Subjects

The naturalistic approach to the second-person standpoint allows us to capture the complexity and particularity of our real interactive, and intersubjective, relationships. As already mentioned, Darwall’s analytical approach avoids this line of reasoning. But a naturalistic approach of the second-person standpoint makes clear that the kind of relationship that binds subjects is not an abstract one that holds equally with any member of the moral community. Rather, it is a particular, emotionally-loaded, relationship which is established with specific persons during our lifetime; and which invests each other with variable degrees of authority to yield particular demands, depending on the situation and the agents involved. For instance, our friends’ demands bind us in a different way than our neighbors’ or any strangers’ ones do.

As Wallace notices, “those who are implicated in a nexus of relational normativity possess a kind of practical authority over the relevant normative relation that uninvolved third parties lack” (Wallace 2007, p. 29). For instance, the victim of a moral transgression has both a “privileged authority to complain” (Wallace 2007, p. 29), and the possibility to consent a behavior against her dignity that would be otherwise considered a transgression. Consequently, “the person who is wronged by you has a privileged basis for complaint against you, an objection to your conduct that is not shared by mere observers to what was done” (Wallace 2007, p. 29). Aiming at our moral psychology, this view seems correct.

Darwall makes sense of this experience through the notions of “obligations of loving relationship” (2016, p. 172), or “duties of relationship” (2016, p. 177). These are moral duties that are shaped by the specific circumstances where they take place, and which are addressed to “a-person-who-happens-to-stand-in-that-specific-putatively-normative-relation” (2006, p. 270). He seems to think, for example, of intergenerational duties: all sons and daughters have certain duties, in virtue of being sons and daugthers, towards their parents. These duties are still moral, because they pass Kant’s test of universalizability and impartiality. We could not conceive a world where victims did not have a special authority towards transgressors; where friends did not have a special authority towards other friends; and where social causes were not given more weight than personally romantic desires. Therefore, Darwall acknowledges the special authority of subjects standing in particular relationships.

Nevertheless, Darwall’s point is rather that blame is assessed not from the victim’s point of view, but from anyone’s, from an impartial standpoint. In his own words, “although resentment is an attitude that can intelligibly be felt only from a victim’s individual standpoint, or from one that identifies with it, blame can be felt from anyone’s standpoint; it entails the representative authority of the moral community” (2018, p. 814). According to Darwall, it is critical that the claims and demands that are addressed in a particular circumstance are justified, and sanctioned from anyone’s point of view, and this entails that its legitimacy is independent of anyone’s particular connection to the situation. Darwall finds inspiration in Adam Smith’s (1759) notion of an impartial spectator. Through this notion he connects the dynamics of particular claims and the Kantian procedural requirement of impartiality as the validity criterion for moral claims. Therefore, even our special obligations towards our friends should be considered as such by anyone who was in our position in relation to them. Hence, as explained in "Introduction" section, Darwall’s project is Kantian at bottom. In Darwall’s morality, the demands that we address to each other must ultimately be ones that could be endorsed from a “perspective that we can all share as free (second-personally competent) and rational” (Darwall 2006, p. 276); they are grounded in our “common authority to make claims on each other” (Darwall 2006, p. 274). In Wallace’s terms, Darwall’s picture of morality is “one on which normative principles get traced in the end to a kind of (hypothetical) collective self-legislation, whereby we make principles normative for ourselves by imposing them on ourselves from a common point of view” (Wallace 2007, p. 32).

However, this common point of view is difficult to sustain within a naturalistic framework. On the one hand, there may not be a unique way to generalize other’s perspectives. There are many situations in life where we relate to others in ways that are not institutionalized, which may be diverse and new, and which may give rise to blameworthy actions without excuse. In these situations we relate to those who harmed us from the point of view of someone harmed by them, because of the particular relationship held with them. These actions matter first of all to us, as people involved in that particular circumstance, and provide us with a distinctive authority over those who harmed us (Corbí 2005). Saying that we could find a description of the situation that would allow us to generalize the demand to any agent in the same circumstances misses a psychological point: it is those involved in the particular relationship that address claims and recognize duties to each other; and it is also them who have a “unique position to alter the normative relations at issue” (Wallace 2007, p. 29) by consenting the kind of behavior that would be otherwise prohibited for the sake of his position as bearer of the violated right.

From this point of view, the practice of blaming takes place first of all within a particular relationship, where participants hold each other accountable depending on their specific stories of relation and affection. In this view, subjects feel bound not by an abstract relationship with any member of the moral community, but by a particular and emotional relationship which is established between specific persons. It is this particular relationship that determines the scope of accountability, and the extension and content of blame. Furthermore, within particular relationships it may also happen that a claim is not recognized as valid, or that an excuse is generated to prevent such a request.

This is nicely expressed by Antoine de Saint-Exupéry in his book *The Little Prince.* Talking about his rose to other roses, the Little Prince acknowledges that *his* rose has special claims over him, which other roses do not have. Specifically, in Chap. 21 he says:

But in herself alone she [*the rose*] is more important than all the hundreds of you other roses: because it is she that I have watered; because it is she that I have put under the glass globe; because it is she that I have sheltered behind the screen; because it is for her that I have killed the caterpillars (expect the two or three that we saved to become butterflies); because it is she that I have listened to, when she grumbled, or boasted, or ever sometime she said nothing. Because she is my rose.

The reason for the distinctive authority that the rose has over the Little Prince is, as he notices, that they have a special relationship; they are friends. Consequently, he has some special duties towards his rose, by virtue of being *that* rose.

The feeling of being bound by the moral norm still comes from the fact that we can be held accountable for non-compliance without excuse. But now there is a difference between the persons that set demands on us as members of the moral community, and those who do it as particular persons that stand in particular relationship with us. Our feeling of being bound by the moral law is enhanced because of the special authority that different persons have on us, due to our relationships with them. For instance, the claim that comes from the person whose foot I stepped into binds me especially because I have a particular relationship with that person as the victim of my transgression. Remarkably, each agent may be part of multiple such relationships within a community, constituting a network of interpersonal links. As we will see in "The Emergence of Group Norms" section, it is this web that helps to explain how a community’s normative common code can emerge and be shared.

## Moral Emotions

Conceived in the naturalized manner we propose, the second-person standpoint is specially linked to moral emotions. First, because moral emotions have a role in our communication of demands, as we explained in "The Second-person Standpoint as a Conceptual Analysis" section. We can react to what another did to us by expressing emotions whose content implicitly involves an appraisal of the particular episode of relation, and in this way, we demand recognition from her. The achievement, or failure, of recognition triggers also specific emotions. Second, moral emotions also have a role in the way we establish and sustain relations to others. Moral subjects (or persons) experience the need to bond affectively and emotionally with particular others, for instance through relations of friendship, trust or love; hence, we need to interact second-personally with others.

Darwall focuses especially on the first point: moral emotions as a way to implicitly address demands or react to other’s demands. He relies on the way Strawson characterized them as “reactive attitudes” (Strawson 1974), to point out that they amount to implicit forms of blaming that assume that agents are accountable for their actions. Reactive attitudes address implicit demands, whereas explicit demands require verbal expression. In Darwall’s account, a reactive attitude is a form of communication which takes place in interpersonal interaction, which is elicited as a response to a person’s behavior, and which seeks to reestablish the recognition and reciprocal respect that participants owe to each other as members of a community of mutually responsible agents. Thus, they entail that both participants must recognize themselves and each other as fully morally responsible agents who are able to participate in adult relationships.

For instance, if I am roaming the streets and suddenly someone pushes me away and does not apologize, I will feel unrecognized as an agent who deserves apologies, and I will probably react to this with indignation. Upon noticing my implicit claim and recognizing it, the agent is expected to repair the situation by expressing regret, or even helping me. This pattern of psychological interaction implicitly involves a basic level of normativity, that is, it demands a right attitude from the other towards me, and from me towards the other.

As explained in "A Naturalistic Approach to the Second-person" section, our naturalistic approach to the second-person standpoint characterizes the psychological competence which ensures understanding, and hence mediates second personal interactions. We interact with particular others by implicitly and mutually attributing mental states in an online, emotionally and intentionally engaged way (Gomila 2001a, 2002, 2015). Moral emotions are an example of this kind of spontaneous, implicit and online interaction. From this point of view, moral emotions constitute an intersubjective means of implicitly addressing claims to others, as Darwall and Strawson notice. But as moral emotions develop in time, they also constitute a story of the relationship, which explains the web of affiliations and preferences from which particular duties derive.

Secondly, moral emotions build bonding relationships with others. Not only are moral emotions a way to communicate a demand, they also give rise to affiliative attachments. I may feel guilty for not having paid enough attention to someone who made me a favor before; I may feel humiliated by somebody that time after time ignores my opinions; or I may feel resentful at somebody’s reluctant way to excuse their transgression, because when the roles were reversed, I felt fully accountable for what I did. Through this kind of sequences of interactions we generate personal preferences, which may become moral norms. Again, a naturalistic approach goes beyond Darwall’s.

As an illustration of this twofold role of moral emotions, consider the following example^1^. Imagine I promised my friend Patrick that I would have dinner with him tonight. On my way to Patrick’s, I see myself involved in a car accident where I am the only one who can help the victim. Although I am still morally obliged to keep my promise, I am also morally obliged to help another in need, especially when nobody else can. So I help that person while assuming that Patrick will feel resented; and I will probably feel guilty, for not keeping my promise. Remarkably, both reactive attitudes are not justified, according to Darwall. As what I did was morally correct, given the circumstances, I am not blameworthy, and hence Patrick’s remorse and my guilt turn out to be misplaced. However, when we take into account the second role of moral emotions, we realize that what I did damaged to some extent my friendship with Patrick. In fact, it would be surprising if I did not feel guilty for failing to keep my promise to him. A similar and historical example of these fitting although unjustified emotional responses is to be found in Primo Levi’s and other survivors’ experiences of shame, and guilt after their liberation from Auschwitz, as if their survival was a treason to their dead fellows (Levi 1986/2017). Although in this case it is an open question whether these moral emotions are actually justified (Corbí 2005), what these examples show is rather that the way we are connected to other people influences which actions and omissions are viewed as cause for a claim of proper respect and recognition; and which emotional reactions are to be expected.

To the limit, it can be said that even if a reactive attitude might be unjustified at the normative level, it is still important to recognize its role in our moral psychology of attachments and affiliations. I would feel confounded if Patrick was not any angry at me for not meeting him for dinner; and he would feel confounded too if I did not show any guilt while telling him that I cannot make it for dinner. My excuse is good and justified, but still we can help being affected by the incident. Given our psychology, feeling those emotions is a natural response; and the lack of those feelings points to emotional distance. We expect from people to respond emotionally to us, according to the quality of our bonding. Part of this mutual responsivity involves addressing and recognizing implicit demands.

At this point, then, our approach also separates from Darwall’s. For Darwall, forms of affective bonding are related to what he calls “attitudes of the heart”. According to him, attitudes of the heart are part of “that aspect of the human psyche through which we are heartened or disheartened, inspired or deflated, encouraged or discouraged, filled with hope and joy or deflated with despair, emptiness, or sadness” (Darwall 2017). They help us bonding together because they seek reciprocity, personal attachment and connection. Some examples of these attitudes are love, trust, gratitude or personal hope. For Darwall, attitudes are not part of morality. They are not part of the accountability domain and hence they are not deontic. They do not put claims on us, because they must be freely given. Therefore, they do not have a function in morality. However, as we have argued, these ways of affiliative bonding do license the sort of moral emotions that we have just described, which implicitly address and recognize second-personal deontic claims.

We humans have a need to establish long-term bonds because it has been evolutionarily adaptive (Cheney and Seyfarth 2008; Seyfarth and Cheney 2012; Tomasello 2016). Starting with the affiliative bonding to our parents, which is related to our long dependence on them in development, we come to establish a variety of affective bonds with others along our lives. This bonding involves prosocial preferences for those we are bonded with, but also sensitivity to their demands on us. It is not that we have abstract prosocial preferences for anyone, but rather we develop particular prosocial preferences for those with whom we come to establish affective, long-term relations. Attitudes of the heart contribute to this bonding through the moral emotions they give rise to. These emotions involve implicit forms of normative assessment, in the form of appraisal. And this is why the precursors of both second-personal relations and morality might be found in this kind of pre-normative bonds, promoted by attitudes of the heart.

In sum, those moral emotions which appear in the context of affectionate relationships, work as a way to address justified demands, as Darwall and Strawson notice; but also as a way to interact second-personally with others, and to connect with them. Indeed, Strawson’s reactive attitudes presuppose a relationship which is interactive and which involves a second-personal way to relate to others. Accordingly, moral emotions illustrate the second-person standpoint because they ensure the commitment that characterizes intersubjective relationships; hence, they become a bridge to morality. From this point of view, morality emerges out of a group of interrelated individuals; and therefore moral emotions become the intersubjective grounds of morality.

## The Limits of the Moral Community

The naturalistic turn of Darwall’s second-person standpoint of morality can also provide an answer to the question about the limits of the moral community. This answer avoids speciesism –a question that can barely be raised within Darwall’s analytical project. Certainly, within the framework of the second-person standpoint of morality, it may be said that the members of the moral community are those who are second-personally competent. Second-personal competence is “the capacity to make demands on oneself from a second-person standpoint: in being able to choose to do something only if it is consistent with demands one (or anyone) would make of anyone (hence that one would make of oneself) from a standpoint we can share as mutually accountable persons” (Darwall 2006, p. 35). In other words, it is the capacity of the subjects “to determine themselves by these [second-personal] reasons” (Darwall 2006, p. 21), and to enter into relations of mutual accountability with other subjects (Darwall 2006, p. 33). Second-personal competence is what makes us subject to moral obligation, and what gives us an authority to make claims and demands of one another as members of the moral community.

Darwall agrees that acting according to second-personal competence requires some psychological capacity for perspective taking and psychological attribution –a capacity that he envisions in terms of “simulation” or “imaginative projection” (Darwall 2006, p. 44–45). It also requires the ability to assess the situation from an involved other’s standpoint, as the precursor to an impartial perspective. Subjects must be able to consider another’s standpoint and “compare the responses that one thinks reasonable from that perspective with the other’s actual responses, as one perceives them third-personally” (Darwall 2006, p. 48). Besides, subjects must be able to regulate themselves by claims, demands, and norms in a spontaneous way, as all these assumptions and dynamics are taken for granted without necessary awareness of them (Darwall 2006). Therefore, the question whether certain individuals count as moral subjects can be reformulated in terms of whether they have the capacity to claim and respond to claims; to recognize another’s authority to claim and being recognized the same authority by others; to hold and being held accountable; and to enter in this kind of second-personal, spontaneous interactions.

In this way, Darwall’s approach offers a way to proceed in order to determine the limits of the moral community: an individual will count as a moral subject if she shows the relevant psychological features described. The question, then, is whether any non-human animal exhibits the same sort of psychological capacities on which such competence depends. Some of them have already been mentioned: perspective-taking, intentional attribution, emotion expression and emotion recognition. This strategy would parallel the one followed by Gomila (2001b), and Gómez (1998), with respect to whether great apes comply, and to what extent, with the conditions of personhood specified by Dennett (1976). However, the difficulty with this strategy when applied to Darwall’s second-personal competence is that the presence of the psychological requirements of such competence may not be sufficient to guarantee the presence of the competence itself. Chimpanzees might have all the necessary psychological processes for second-personal interaction, but still not be second-personally competent, in Darwall’s sense, i.e., moral beings.

An alternative strategy is to directly examine the evidence of second-personal competence in non-human animals. If being a member of the moral community consists in addressing demands to one another through implicit emotional reactions, which already entail some form of normativity, then the task is to find out whether any species exhibit this kind of sensitivity to moral claims, through the appropriate emotional reactions. Taking this approach, the research groups led by Kristine Andrews (Andrews 2009; Vincent et al. 2019), Mark Bekoff (Bekoff 2004; Pierce and Bekoff 2012) and Frans de Waal (Brosnan and de Waal 2003; de Waal 1996; Waal 2006, 2014) have argued that non-human primates do relate through normative expectations, and do react with anger and rage when those normative expectations are transgressed. However, this interpretation is controversial. Michael Tomasello and his group are more skeptical about non-human animals’ second-person competence. According to them, we have enough negative evidence to deny any normative capacity in non-human animals. The behaviors that the first group take to show resentment are also compatible with disappointment and frustration (Engelmann et al. 2017; Engelmann and Tomasello 2017; Tomasello 2016). Regardless of the way the evidence turns out, the point here is that Darwall’s notion offers a fruitful path to study the question of the limits of the moral community.

## The Emergence of Group Norms

Another important question, both in Darwall’s analytical project and in our naturalistic interpretation of it, is the relation between the demands addressed within the dyadic structure of second-person relationships, and the explicit norms and codes the social groups of humans develop in time.

In Darwall’s project, there is no difference between the valid demands in the dyad, and the universally valid ones; the categorical imperative that justifies the demands, according to their universalizability and impartiality, is already assumed in the intersubjective relation. For the interaction to occur in the first place, participants need to be committed to a categorical and universal principle (de Maagt 2018). If I engage in a second-personal relation with someone else, I assume that the other will comply with my demands only if they sees them as justified. In Darwall’s words, “you and she commonly presuppose that she can freely comply if she finds your request or demand one she could not reasonably reject, regardless of what she desires or how strongly she desires it” (Darwall 2006, p. 245). Yet in everyday practices, an addressee might not recognize the demands of an addresser. For instance, my demand as a customer of being attended in English might not be recognized by the shop-assistant, who might consider that my claim violates his dignity as a French speaker. Or, as Corbí (2005) forcefully describes, a victim might not be recognized by a torturer; i.e. the torturer might deny the victim’s experience of the harm that they themselves is causing. Hence, both the shop-assistant and the torturer might see the demands of their customers, and victims respectively unjustified. This lack of agreement about the validity of demands is possible within a group; much more so when the relationship is established between members of groups that disagree about the relevant norms in the first place.

To explain what justifies a demand, Darwall provides an analytical answer, resorting to a version of Smith’s impartial spectator (1759). According to Darwall, a demand is justified if any member of the moral community would accept it. Justified blame is addressed as if from an impartial third-party. Consequently, although second-personal demands are described as happening in the dyad, they have already universalizing tendencies (Darwall 2018); they are expressed as if any member of the moral community could legitimately address it to another member whoever. Therefore, Darwall does not need to explain how the norms accepted within the dyad extend to the other members of the moral community. In Darwall’s view, the universality of the demands is prior to the dyad. As already said, the validity of the demand does not depend on its being addressed.

As already pointed out, though, the assumed impartial spectator’s view cannot be taken for granted. This strategy faces two sorts of related problems: there is no such an impartial spectator’s perspective; and different communities can have different moral codes. First, as we mentioned in "Special Obligations Towards Particular Subjects" section, there is no such a generalized or impartial perspective. No individual, as rational as they might be, can adopt such generalized, or impartial, perspective and conclude whether a claim is justified or not. Second, if there were such a generalized perspective, a unique set of demands could be justified. Yet, in practice, what happens is that different communities find legitimate different sets of demands, because they accept different moral codes.

Both issues cannot be solved within Darwall’s approach. A naturalist approach, on the contrary, offers a way to raise the question of how reciprocal demands become valid through the group consensus. Allan Gibbard dealt with the question of the emergence of group norms, and the possibility of moral disagreement, from such naturalist standpoint. According to Gibbard (1982, 1989), moral norms emerge out of interaction with others. When we interact, we stick to our positions and avow them; but we also discuss, and look for a consensus. As a consequence of this interaction, “a group will form a community of judgment, and different groups may form different communities of judgment, at odds with each other” (Gibbard 1989, p. 177). In the different communities, there will be discrepancies on local norms; whereas there will be consensus on some restricted topics “on which one needs agreement” (1989, p. 179). Accordingly, beliefs about justice will be “beliefs about what the consensus will be on what is just”(Gibbard 1982, p. 42).

The naturalized second-person approach can still resist this view of normative consensus through explicit argument by pointing out that there is no need to foresee a communitarian process of explicit normative debate and consensus. Given that each agent can establish dyadic interactions with many others, the web of dyadic interactions among members of the community that exchange their roles as addressers and addressees is bound to be consistent in their sanctioning demands. Thus, it emerges a sort of collective equilibrium that needs not be reflexive. The members of a community will share their emotional repertoire and each member will exchange roles in their respective interactions with other members of the community. The global dynamics of interaction will require similar responses to similar situations, thus emerging a normative consensus without an explicit debate. Once this equilibrium is reached, the authority of each subject to address demands based on reasons is warranted by the authority of the group and, finally, by the moral community, who has the role of accepting and approving demands, and requiring respect of members, in cases of disagreement (Corbí 2005).

A similar process may unsue among communities with different normative standards that come to interact. The interaction among members of each community will require some common ground, both to make them reciprocally understandable –through second person intentional attributions-, and to validate or not the demands that might emerge. In this case, though, there is no guarantee that a consensus will be reached. The communities might rather prefer to restrict their interactions, or the most powerful community might impose their norms or even simple domination.

From this perspective, the objectivity of moral norms can be compared to the objectivity of the norms of grammar^2^. Grammar norms of a language are objective in the sense that we cannot make them up. Yet they are not mind-independent because they depend on the minds of the speakers of that language. The same holds for moral norms: they are objective in the sense that we as particular individuals cannot change them; but they are not mind-independent because they depend on the dynamics of intentional interaction of the agents. Thus, intersubjective interaction allows us to account for both the presence of group norms, and the disagreement about them among different communities.

## Conclusion

Darwall’s second-person standpoint of morality is undoubtedly a valuable contribution. Its central idea is the attempt to ground moral obligation in mutual recognition between subjects. Yet its analytical and Kantian nature prevents it from developing its potential as an account of our moral psychology. In this paper, we have developed Darwall’s proposal from a naturalistic standpoint, and argued that such understanding of its central idea helps explain a variety of features of morality: the motivational power of moral judgments; the special obligations we recognize towards particular subjects; the intersubjectivity involved in moral emotions; the limits of the moral community; and the emergence of moral, group norms.

In any case, Darwall helps to make clear that morality is not in the business of strategic self-interest, but the key to the significant interpersonal relationships that make our lives unique and valuable.

This work was supported by the Spanish Ministry of Education, Culture, and Sports under grant FPU14/01186; and by the Spanish Ministry of Economy, Industry and Competitiveness under Grant FFI2017-86351-R. We thank Stephen Darwall for comments on earlier drafts.

## Data Availability

Not applicable.

## Footnotes

1 We are indebted for a similar example to Stephen Darwall.

2 We are indebted for this example to Shelly Kagan.
